# Influence of Impurities on the Process of Obtaining Calcium Carbonate during the Processing of Phosphogypsum

**DOI:** 10.3390/ma15124335

**Published:** 2022-06-19

**Authors:** Igor Pyagai, Olga Zubkova, Rodion Babykin, Maria Toropchina, Roman Fediuk

**Affiliations:** 1 Department of Mineral Raw Materials Processing, Saint-Petersburg Mining University, 199106 St. Petersburg, Russia; igor-pya@yanix.ru (I.P.); zubkova-phd@mail.ru (O.Z.); rodion_babykin13@mail.ru (R.B.); toropchina_maria@bk.ru (M.T.); 2 Polytechnic Institute, Far Eastern Federal University, 690922 Vladivostok, Russia

**Keywords:** environment, phosphogypsum processing, calcium carbonate, carbonization, associated components

## Abstract

The article is devoted to the study of the influence of residual sulfuric and phosphoric acids on the process of processing large-tonnage phosphogypsum (PG) waste into calcium carbonate. In the Russian Federation, about 10 percent of existing phosphogypsum waste is processed into construction materials. Acidic impurities (phosphoric and sulfuric acids) in their composition are an obstacle to the use of phosphogypsum for the production of binders. This study finds that impurities also reduce the chemical activity of phosphogypsum. At the same time, the paper focuses on the potential of phosphogypsum for the production of calcium carbonate. This article investigated the amount of impurities in phosphogypsum. The results show that during automatic washing of phosphogypsum, losses are approximately 30–35 wt. %. It was also found that phosphogypsum by 22% consists of impurities of phosphoric and sulfuric acid. These acids are characteristic waste products of extraction phosphoric acid (EPA) production. By ASTM C471M-20ae1, the content of calcium sulfate dehydrate and hemihydrate before and after washing was determined. A thermodynamic calculation of the proposed interaction of phosphogypsum with carbonates showed that the characteristic reaction is possible. The conversion process of phosphogypsum to get the corresponding calcium carbonate was carried out at 70 °C. Data on the chemical composition of the reaction products, obtained by X-ray fluorescence analysis on a Shimadzu EDX-7000 spectrometer, showed that some reactions proceed incompletely and need the selection of optimal conditions. The potential commercial benefits of processing phosphogypsum by carbonization were defined for products such as calcium carbonate or its derivatives.

## 1. Introduction

The intensification of agricultural production is constantly increasing the amount of consumption of various types of fertilizers. A special role in the restoration of soil fertility is played by phosphorus-containing mineral fertilizers, through the use of which the phosphate regime of soils is regulated. To date, most phosphorus-containing mineral fertilizers are produced based on extractive phosphoric acid (EPA). Calcium sulfate dihydrate (CaSO_4_∙2H_2_O) or calcium sulfate hemihydrate (CaSO_4_∙0.5H_2_O) is produced by the sulfuric acid method of EPA production. Phosphorus compounds are part of the impurities (phosphoric and sulfuric acids) that give the name to the waste phosphogypsum (PG) or phosphohydrate. However, both secondary material resources are referred to as phosphogypsum when considering transportation, storage and use. From 4.2 to 6.5 tons of phosphogypsum is formed in the process of producing 1 ton of P_2_O_5_ to EPA (in terms of dry divalent calcium sulfate), depending on the quality of the processed phosphate rock [1,2].

The problem of using phosphogypsum for national economic purposes began to appear before the researchers were already at the stage of developing processes for obtaining EPA. Nevertheless, the widespread and cheap nature of gypsum hindered the introduction of developing technologies for processing phosphogypsum.

On the other hand, world experience shows that phosphogypsum can be successfully and efficiently used and processed to obtain high-quality products [3,4]. In 2013, the total share of phosphogypsum used worldwide was at least 14.1% of the total material produced.

In Russia, about 1% of phosphogypsum is processed, and the trend of constant growth of the production capacity of EPA leads to the fact that annually phosphorus-containing waste is formed in the amount of 140–200 million tons. This is explained by the fact that under current conditions, it is more economical to store phosphogypsum than to use and comprehensively recycle it. Therefore, in Russia, the problem of phosphogypsum utilization is not solved, despite the fact that it can be used as a binder in road construction [5,6,7]. This type of disposal cannot be considered large-scale recycling of these wastes. Moreover, transportation of phosphogypsum is difficult, even over medium distances, due to its high hygroscopic and easily movable sludge.

The process of processing phosphogypsum into new marketable products is relevant in the 21st century. The use of phosphogypsum, accumulated in the dumps of industries, will minimize the technogenic impact on the environment, and it will also contribute to reducing the extraction of minerals (gypsum and anhydrite) from the subsurface and as a result, will reduce the amount of deposited chemically precipitated calcium sulfate. It should be noted that the solution to the problem of using chemically precipitated gypsum is only possible with a comprehensive approach.

The use of phosphogypsum waste is promising in that it will reduce the cost of construction materials and improve their physical and chemical properties. For this reason, scientists are conducting various studies in this direction.

Studies on road construction in Russia [5,6,7] showed positive results, but this technology also has not been widely used. The work of Tian T. [8] and Ding W. et al. [9] (China) discusses the use of phosphogypsum as a raw material for concrete production. The results show that the optimum content of PG was 45–55%, the ratio of cement to HGBFS—1%, quicklime—4% and sulphoaluminate cement—2%. 

According to the literature review, road construction technology does not take into account the content of residual acids that leak into the soil and lead to adverse effects.

Zhang W. et al. [10] consider the use of phosphogypsum and CO_2_ for the carbonization process. The carbonization has a yield of 90% but has not found any industrial application. Ma J. et al. [11] and Chen Q. et al. [12] discuss the carbonization process of phosphogypsum with ammonium carbonate. The result is CaCO_3_ of very high purity.

Papers [13,14] described techniques for studying calcium carbonate. In paper [13], a halochrome TF PLPt sensor was used which scans in real-time the interaction of alkaline medium and calcium carbonate in concrete. In [14] the effect of gypsum waste types on the properties of alkaline slag cement paste was studied using XRD, TG-DTA and SEM-EDS methods. Some of their research methods are noteworthy and will be taken into account when studying the acid effect of calcium sulfate during conversion to calcium carbonate. 

The authors of these articles obtained good results in obtaining calcium carbonate. The obtained calcium carbonate has a finely dispersed structure, which makes it difficult to process PG.

Paper [15] presents the effects of aluminum sulfate and quicklime/fluorgypsum ratio on the properties of calcium sulfoaluminate cement-based double liquid grouting materials. Article [16] researched the effects of calcium bicarbonate on the properties of ordinary Portland cement paste. Paper [17] characterizes the recycling of waste seashells with Portland cement towards sustainable cementitious materials.

The extraction of rare earth elements from phosphogypsum is worth mentioning separately. In works [18,19,20] methods of rare earth elements (REE) extraction from anthropogenic raw materials, including phosphogypsum, are considered. This kind of processing could solve the problem with environmental load and REE production.

The extraction of REE from phosphogypsum requires large energy inputs with a small yield of the target product, which is not feasible for enterprises.

The purpose of this study is to identify acid impurities in waste phosphogypsum and their impact on processing phosphogypsum. This study also considered the issue of increasing the purity of the initial phosphogypsum, to improve the efficiency of processing on the example of obtaining CaCO_3_.

## 2. Materials and Methods

The object of the study was selected dump phosphogypsum from the industrial site (Phosagro, Volkhov).

This phosphogypsum is a powder of gray-white color, the mass fraction of the main substance in the recalculation of calcium sulfate—60%. The salts Na_2_CO_3_, K_2_CO_3_ and (NH_4_)_2_CO_3_ were used to conduct studies on the effect of the acidic environment of phosphogypsum. The research was based on the following reactions (1)–(3):

CaSO_4_ · 2H_2_O + Na_2_CO_3_ = CaCO_3_↓ + Na_2_SO_4_ + 2H_2_O (1)

CaSO_4_ · 2H_2_O + K_2_CO_3_ = CaCO_3_↓ + K_2_SO_4_ + 2H_2_O (2)

CaSO_4_ · 2H_2_O + (NH_4_)_2_CO_3_ = CaCO_3_↓ + (NH_4_)_2_SO_4_ + 2H_2_O (3)

These reactions were carried out after each of the series of washings. After filtration and drying, the resulting precipitate was examined for CaCO_3_ content, in accordance with two methods: X-ray fluorescence analysis and back titration.

*Research stages*: washing phosphogypsum with water, determining the residual content of acids, the effect of acids on reactivity, calculating the possibility of carrying out reactions under various conditions, researching the composition and properties of washed and unwashed phosphogypsum, conducting reactions to obtain calcium carbonate, researching the reaction product and the resulting salts.

### 2.1. Washing Phosphogypsum

The phosphogypsum was washed in a jacketed glass HEL mono-reactor. The weight of the sample was 60 g per 1 L of distilled water. The process was performed for 1 h, at a constant temperature of 70 °C and stirring at 200 rpm. Measurements of pH of washing solution with the determination of H_3_PO_4_, H_2_SO_4_ acids by potentiometric titration [21], and weighing of pre-dried powder were carried out after each washing procedure. 

### 2.2. Chemical Composition of the Reaction Products 

Chemical composition of the reaction products was determined by XRD analysis by an EDX-7000 spectrometer (Shimadzu, Tokyo, Japan).

In washed phosphogypsum, the content of divalent and semi-aqueous calcium sulfate was determined in accordance with ASTM C471M-20ae1.

### 2.3. Determination of Divalent Calcium Sulfate 

It was determined by the following procedure: a small amount of crushed and dried to constant weight phosphogypsum was placed in the calcined crucibles, weighed to constant weight, and for 1 h samples were calcined at 400 °C in a muffle furnace, then cooled in the desiccator and weighed again.

### 2.4. Determination of Semi-Aqueous Calcium Sulfate

To determine semi-aqueous calcium sulfate, gypsum dough was mixed with the addition of water in a 1:1 ratio in a preweighed cup. After solidification, the phosphogypsum was placed in a desiccator for 3 h at 60 °C, cooled and weighed.

### 2.5. Reactivity Studies of Washed Phosphogypsum

After the phosphogypsum washing process in the mono-reactor, reactions (1)–(3) were carried out in an automatic parallel HEL reactor, for 1 h, at T = 70 °C and a stirring mode of 200 rpm using a magnetic stirrer. The resulting reaction products were then filtered using a vacuum filter in order to separate the precipitate from the solution. The first product of the reaction was dried and weighed, and the content of CaCO_3_ by titrimetric method was determined in accordance with ASTM C51-71. The second reaction product, salts, was evaporated at 100 °C to a crystalline state.

### 2.6. Determination of Calcium Carbonate Content by Reverse Titration 

Lime material with a mass of 1 g was placed in a 250 mL conical flask. The lime material was moistened with 10 mL of water, 20 mL of 1 N hydrochloric acid and 30 mL of distilled water were added. The flask with the solution was covered with glass and brought to a boil. The solution was boiled for 5 min, and then 50 mL of hot water was added. Then 4–5 drops of phenolphthalein solution were added to the flask and the titration was carried out with 0.25 N NaOH solution until a faint pink coloration appeared.

## 3. Results and Discussion

### 3.1. Determination of the Chemical Composition of Phosphogypsum from Production Sites in Russia

The chemical composition of phosphogypsum was obtained by XRD analysis. The chemical composition of phosphogypsum is shown in Table 1.

It can be concluded from Table 1 that phosphogypsum can be used for the production of binders and concretes, as proved by the low content of impurities (SiO_2_ and Fe_2_O_3_). The acidic impurities do have a negative impact on the processing of PG. It is desirable to wash phosphogypsum during the processing stages to remove acidic soluble impurities.

From the data, we can also conclude that the chemical composition of PG does not depend on the place of extraction of raw materials for fertilizer production.

### 3.2. Thermodynamic Calculations of Reactions

**Figure 1:**
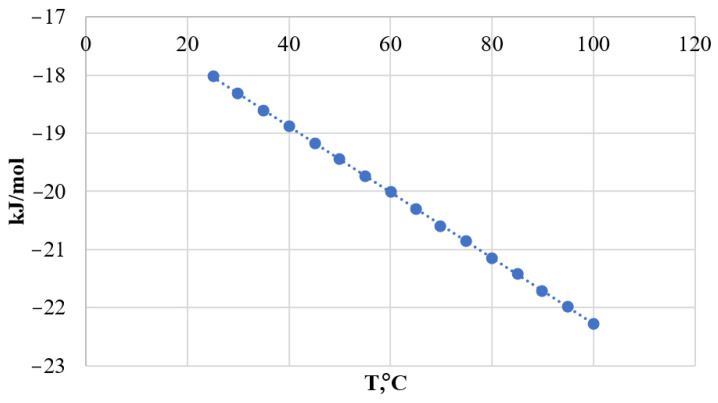
Dependence of Gibbs energy change on temperature.

The change in Gibbs energy from the standard entropy and heat effect was calculated (Table 2) [22,23]. The dependence of the change in Gibbs energy on temperature, using the reaction with K_2_CO_3_ as an example, is shown as a graph in Figure 1.

From the calculations, we can conclude that reactions (1) and (2) are possible under normal conditions without heat and pressure increase. Obtaining products by reaction (3) is not so effective, which may be due to the course of side reactions (reaction (4)):

(NH_4_)_2_CO_3_ = 2 NH_3_ + CO_2_ + H_2_O (4)

Furthermore, Table 2 shows that reaction (3) is more dependent on temperature. It is advisable to study reaction (3) by changing not only the temperature but also the pressure, on appropriate laboratory equipment, for example, in an autoclave [24,25,26].

The graph shows that under normal conditions (T = 273.15 K, P_atm_ = 760 mm∙Hg) the change in Gibbs energy is less than 0. The negative value of the Gibbs energy change, ranging from −17 to −58 kJ/mol indicates the possibility of the reaction of formation of calcium carbonate in the decomposition of phosphogypsum.

The change in the enthalpy of the reactions showed that the reactions using Na_2_CO_3_ and K_2_CO_3_ are exothermic. Conversion will proceed with heat release. These processes do not need additional equipment in the scheme of technological lines of phosphogypsum utilization. At the same time, the reaction (NH)_2_CO_3_ is endothermic and requires additional heat.

### 3.3. Determination of Acid Content

**Figure 2:**
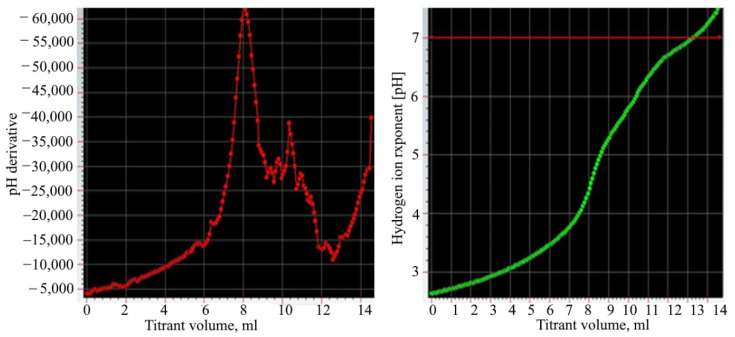
The differential and integral titration curves.

After washing the phosphogypsum, potential titration was carried out. Figure 2 shows differential and integral titration curves. These curves were obtained by titrating the acidic effluent with 0.1 N NaOH solution.

The content of sulfuric and phosphoric acids was calculated from the titration curves. The first point of equivalence corresponds to the neutralization of all sulfuric and phosphoric acids. The second point shows the neutralization of the second hydrogen ion of phosphoric acid. Separate titration of the first and second hydrogen ions phosphoric acid is possible due to a large difference in the values of the ionization constants of phosphoric acid in the first and second steps. This is because the titration results in a one-substituted and then a two-substituted salt of sodium phosphate. The substitution of the third hydrogen ion of the acid occurs without a visible titration jump due to the very small value of the ionization constant [27,28,29].

**Figure 3:**
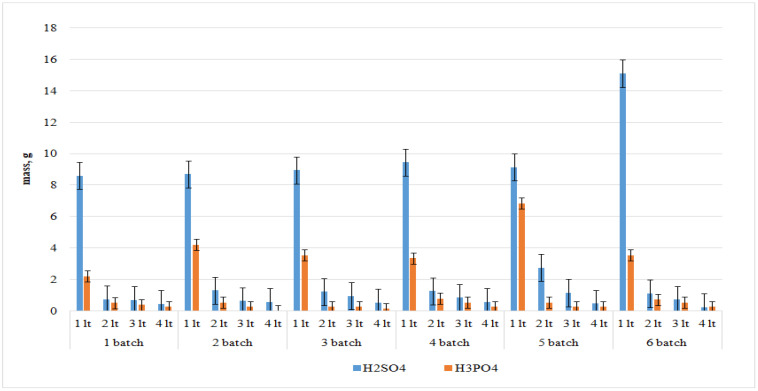
Mass of sulfuric and phosphoric acids in water after a series of phosphogypsum washing with water.

Figure 3 shows the results of the acid mass after washing PG depending on the washing batch. In each batch, 60 g of spent PG were weighed and washed according to the stoichiometric calculation for each reaction considered. It can be concluded that after the washing procedure about 13 g of sulfuric and phosphoric acids are washed out of 60 g of phosphogypsum. These acids represent 22% of the weight of the phosphogypsum leached, in total.

The pH of water was measured after each washing procedure. The average values were: 2.45 to 2.67 after the 1st wash; 3.26 to 4.25 after the 2nd wash; 4.25 to 5.26 after the 3rd wash; and 4.83 to 6.6 after the 4th wash. 

Removal of soluble acid-containing impurities is observed when phosphogypsum is washed with water. Studies show that phosphogypsum storage without washing has a significant negative impact on the environmental situation during heavy rains and snow melting.

### 3.4. Determination of the Chemical Composition after a Series of Washes

According to the results of the chemical analysis the oxides content was, mass %: CaO—34.89; SO_3_—59.89; SiO_2_—3.17; SrO—2.17. During the study, it was found that the mass ratio of insoluble substances in phosphogypsum practically does not change. According to the obtained data on the chemical composition, we can conclude that only acids are washed out of phosphogypsum.

### 3.5. Determining the Grade of Gypsum

Table 3 shows the results of determining the content of dihydrate (CaSO_4_∙2H_2_O) and hemihydrate calcium sulfate (CaSO_4_∙0.5H_2_O) after washing the phosphogypsum by gravimetrical method.

The results from Table 3 show that the content of one modification of calcium sulfate depends on the content of another. The percentage of calcium sulfate dihydrate is higher than that of semi-aqueous calcium sulfate. According to ASTM C471M-20ae1, the analyzed washed phosphogypsum corresponds to grade 4 gypsum [30,31].

### 3.6. Results of the Influence of the Acid Residue on the Yield of CaCO_3_

Table 4 shows the yield of calcium carbonate by titrimetric analysis.

According to the data obtained, it follows that the yield of calcium carbonate directly depends on the content of the remaining acids in phosphogypsum. For the reaction with sodium carbonate (reaction (1)), the content of calcium carbonate in washed phosphogypsum is 1.15 times higher than in acid-containing phosphogypsum. For the reaction with potassium carbonate (reaction (2)), the same value was 1.2 times, respectively.

Examination of the sediment by laser diffraction showed that the coarseness of the CaCO_3_ ranged from 0.2 to 5 nm for all obtained samples [29].

Evaluation of the pore size in the synthesized CaCO_3_ particles was also carried out by the method of adsorption and desorption of nitrogen (the Brunauer–Emmett–Teller method, BET). The BET method showed that the surface area of particles having a diameter of 500 ± 90 nm, is 16.1 m^2^/g, and the particles themselves are mesoporous [32,33].

### 3.7. Microstructure Analysis

**Figure 4:**
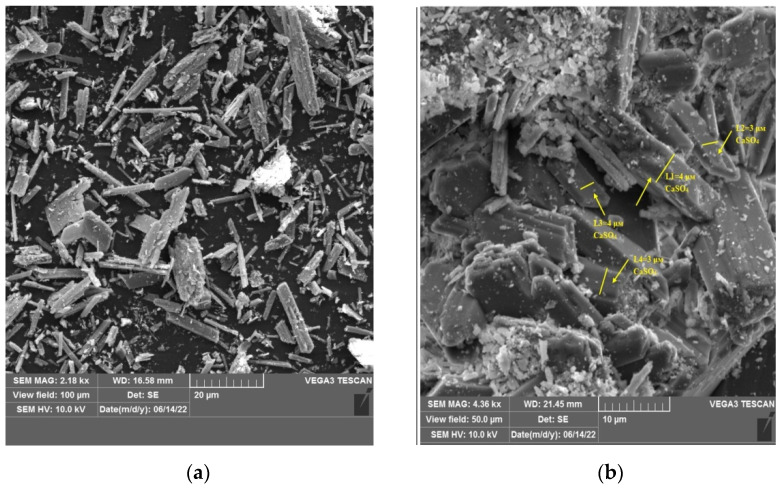

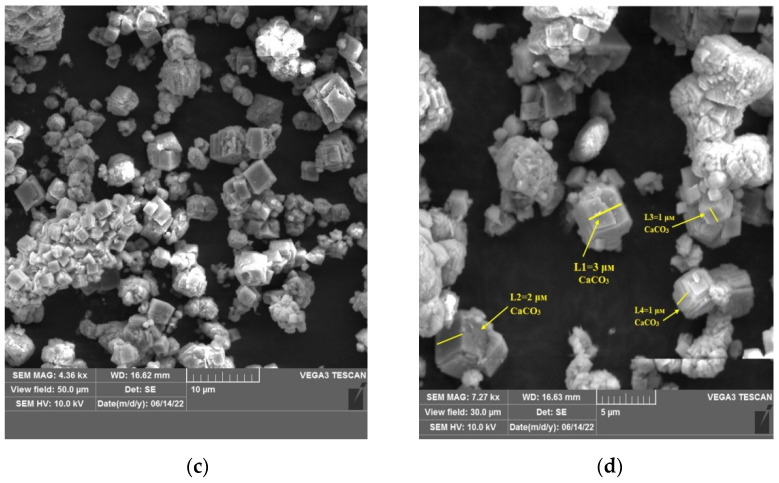
SEM micrograph of (**a**,**b**)—watered phosphogypsum, (**c**,**d**)—calcium carbonate obtained in the course of the conversion.

To confirm the particle size of the obtained calcium carbonate and the completeness of the conversion studies of phosphogypsum (calcium sulfate) washed from residual acids and the obtained sample of calcium carbonate with particle size fixation were performed (Figure 4).

In the course of investigations on a scanning electron microscope, images of calcium sulfate and calcium carbonate crystals were obtained. The needle shape of the crystals corresponds to washed phosphogypsum; the cubic shape of the crystals corresponds to calcium carbonate. In confirmation of the laser diffraction studies, the size of the crystals was measured for calcium sulfate was 3–4 microns, for calcium carbonate 1–2 microns, respectively.

### 3.8. Determination of the Chemical Composition of Reaction Products

Table 5 presents the results of the chemical composition of the products. These products were obtained in the course of reactions 1–2 of pure phosphogypsum.

According to Table 5, the reaction with potassium carbonate at 70 °C is incomplete, as evidenced by the residual content of K_2_O and SO_3_ in product 1. Consequently, this reaction requires the selection of optimal conditions for the complete reaction, so that the product of this reaction is marketable CaCO_3_ and K_2_SO_4_. The data obtained from the reaction with sodium carbonate indicate that the reaction proceeded completely. Despite the small amount of Na_2_O in the calcium carbonate compared to the amount of K_2_O. Reactions 1 and 3 require the selection of conditions [34,35].

**Figure 5:**
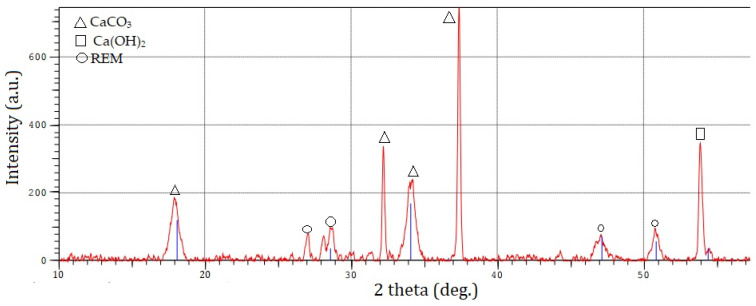
X-ray pattern of calcium carbonate.

The chemical compositions of calcium carbonate determined by X-ray fluorescence are shown in Table 6. The minerals present in phosphogypsum were determined by the X-ray diffraction method. As shown in Figure 5, the samples contain calcium carbonate (CaCO_3_) and calcium hydroxide (Ca(OH)_2_) [35,36].

## 4. Conclusions

The basis of the study is the choice of the direction of effective integrated processing of raw materials of man-made origin. The implementation of such solutions contributes to the rational use of the mineral resource base of the country and improves the efficiency of its reproduction, as well as reduces environmental pollution.

Conducting the conversion reaction of phosphogypsum without preliminary preparatory operations showed a low yield of CaCO_3_, despite the absence of thermodynamic bans. Acidic impurities contained in phosphogypsum reduce its chemical activity because the residues of sulfuric and phosphoric acids also interact with the cations of the introduced carbonates, which prevents the target conversion reaction.

By developing special regimes of washing of initial phosphogypsum it was possible to increase the reactivity of PG by 1.2 times by removing acidic impurities. The yield of CaCO_3_ was achieved up to 70.6% in the reaction with sodium carbonate and 65.0% with potassium carbonate. The washed phosphogypsum was analyzed according to ASTM c412M-20ae1 standards by the following methods: photometry, weight loss, etc. The results obtained indicate that the obtained laboratory sample of calcium carbonate can be used in the construction industry and in the paper industry as a bleaching agent.

Further study of the issue of phosphogypsum conversion with obtaining calcium carbonate will have the aim of obtaining calcium carbonate of higher purity.

## Figures and Tables

**Table 1:** The chemical composition of phosphogypsum from different storage dumps in Russia.

No.	Phosphogypsum Storage Production Sites	Oxide Content, wt.%							
		CaO	SO_3_	SiO_2_	SrO	BaO	Fe_2_O_3_	K_2_O	REM
1	Kingisepp	35.59	61.33	1.37	1.30	0.11	0.08	0.05	0.16
2	Balakovo	33.36	60.21	3.60	1.91	0.11	0.09	0.14	0.57
3	Cherepovets	34.35	60.81	2.01	2.17	0.16	0.11	0.09	0.27
4	Volkhov	36.41	58.72	1.62	2.43	-	0.01	0.11	0.69

**Table 2:** Dependence of Gibbs energy change on temperature.

No. Reaction	T, °C	∆G, kJ/mol	∆H, kJ/mol
No.1 (+Na_2_CO_3_)	25	−25.9	−37.0
	50	−26.0	
	100	−26.2	
	500	−27.8	
No.2 (+K_2_CO_3_)	25	−55.0	−65.3
	50	−55.2	
	100	−55.5	
	500	−58.0	
No.3 (+(NH_4_)_2_CO_3_)	25	−17.8	166.7
	50	−18.6	
	100	−20.3	
	500	−33.9	

**Table 3:** The content of dehydrate and hemihydrate CaSO_4_ in phosphogypsum.

Sample Name	Content CaSO_4_·2H_2_O, %	Content CaSO_4_·0.5H_2_O, %
Pure reagent	93.18	12.0
Washed phosphogypsum	66.9	50.7
Unwashed phosphogypsum	81.12	22.2

**Table 4:** The yield of the finished product CaCO_3_ (%) depending on the acid residue of phosphogypsum.

No. Reaction	Content CaCO_3_, %	
	Acid-Containing Phosphogypsum	Washed Phosphogypsum
1	61.4	70.6
2	52.5	65.0

**Table 5:** Chemical composition of the leaching reaction products of washed phosphogypsum.

No. Reaction	No. Reaction Products	Chemical Composition, wt. %					
		CaO	SO_3_	SiO_2_	SrO	K_2_O	Na_2_O
1	1 product *	**CaCO_3_**					
		90.8	1.8	0.4	4.6	-	*0.9*
	2 product **	**Na_2_SO_4_**					
		-	64.7	-	-	-	33.2
2	1 product	**CaCO_3_**					
		74.8	6.6	8.5	5.1	*3.2*	-
	2 product	**K_2_SO_4_**					
		-	44.9	-	-	53.7	-

* 1 product is CaCO_3_. ** 2 product is the salts after the reactions Na_2_SO_4_ and K_2_SO_4_.

**Table 6:** Chemical composition (wt. %) of calcium carbonate laboratory samples.

Material	CaCO_3_	SrCO_3_	SO_3_	Na_2_O	P_2_O_5_	SiO_2_	REM
Calcium carbonate	90.8	4.6	1.8	0.89	0.52	0.44	0.95
